# Metabolic Syndrome and Its Components Have a Different Presentation and Impact as Cardiovascular Risk Factors in Psoriatic and Rheumatoid Arthritis

**DOI:** 10.3390/jcm12155031

**Published:** 2023-07-31

**Authors:** Fabiola Atzeni, Laura La Corte, Mariateresa Cirillo, Manuela Giallanza, James Galloway, Javier Rodríguez-Carrio

**Affiliations:** 1 Rheumatology Unit, Department of Experimental and Internal Medicine, University of Messina, 98125 Messina, Italy; 2 Centre for Rheumatic Disease, Kings College London, London WC2R 2LS, UK; 3 Area of Immunology, Department of Functional Biology, Faculty of Medicine, University of Oviedo, 33006 Oviedo, Spain; rodriguezcjavier@uniovi.es; 4 Instituto de Investigacíon Sanitaria del Principado de Asturias (ISPA), 33011 Oviedo, Spain

**Keywords:** metabolic syndrome, arthritis, psoriatic, arthritis, rheumatoid, heart disease risk factors

## Abstract

Patients with chronic inflammatory arthritis have a higher cardiovascular (CV) risk than the general population. Traditional CV risk factors are clearly implicated, while the impact of metabolic syndrome (MetS) is less defined. The aim of this study was to compare MetS prevalence and impact on the CV risk in psoriatic arthritis (PsA) versus rheumatoid arthritis (RA). A retrospective analysis of real-world data of PsA and RA patients referred to a rheumatology clinic was conducted. The following data were extracted and compared: demographic data; clinical data; presence of traditional CV risk factors and MetS. Univariate and multivariate models were used to compare the impact of MetS and its components in patients with PsA versus RA. Overall, 170 patients were included (PsA: 78; RA; 92). The two groups differed significantly in mean age, disease duration, and presence of MetS, while other variables were comparable. Univariate and multivariate analysis identified distinct predictors of MetS in PsA (hypertension) and RA (dyslipidemia). The history of CV events was similar in the two groups. Predictors of CV events were MetS and most of its components in PsA, while dyslipidemia was the strongest predictor in RA. These associations were stronger in PsA than in RA. In conclusion, the impact of MetS and its components is different in PsA and RA. The association of these risk factors with CV events is stronger in PsA than in RA. This suggests the implication of different mechanisms, which may require distinct strategies for the prevention of CV events in PsA and RA.

## 1. Introduction

Rheumatoid arthritis (RA) is associated with an increased risk of cardiovascular (CV) morbidity [1,2,3,4]. RA is now widely recognized as an independent risk factor for CV disease, similar to diabetes [5,6]. An elevated risk of CV and cerebrovascular events compared to the general population has also been reported for psoriatic arthritis (PsA) [2,7]. The increased CV risk associated with RA and other chronic inflammatory conditions is likely caused by the complex interplay of traditional CV risk factors, chronic systemic inflammation, and side effects related to the use of certain antirheumatic medications [8,9]. Although a similar figure has been proposed for PsA, to what extent these arthropathies differ is yet to be clarified.

Traditional CVD risk factors include older age, male gender, smoking, hypertension, dyslipidemia, and diabetes [10]. A specific clustering of metabolic and CV factors, known as metabolic syndrome (MetS), has also been found to substantially increase the risk of CV events [11]. The impact of MetS on the CV risk in inflammatory arthritis is yet to be clarified.

Several studies have investigated the epidemiology of traditional CV risk factors and MetS in patients with RA and PsA [9,10,12,13,14,15]. A study based on the data from a US health insurance claim database estimated the prevalence of CV risk factors during the 12 months before diagnosis and their incidence rates during follow-up in patients with RA, PsA or psoriasis [12]. The prevalence was elevated in all diseases and ranged from 17% to 20% for hypertension, 6% to 8% for diabetes mellitus, 10% to 12% for hyperlipidemia, and 4% to 6% for obesity; incidence rates of CV risk factors were also high [12]. The largest study covering this topic, using a medical record database from United Kingdom, revealed a higher prevalence of all CV risk factors in PsA patients, whereas only diabetes and obesity were found to be increased in RA. The incidence of new diagnosis of hypertension, hyperlipidemia and diabetes mellitus was elevated in both RA and PsA [13]. However, MetS occurrence has received less attention. A recent meta-analysis compared MetS prevalence in patients with PsA versus RA and found that PsA patients were 1.6 times more likely to present with MetS than their RA counterparts [14]. Nevertheless, whether MetS clinical presentation and their components were comparable across conditions is unclear.

Overall, the epidemiologic evidence confirms the association between chronic inflammatory joint diseases and traditional CV risk factors and suggests that this association may differ among diseases [12,14,15]. These differences need to be further investigated as they may have an impact on the prevention and management of CV disease in RA and PsA. In fact, according to the 2015/2016 European Alliance of Associations for Rheumatology (EULAR) recommendations for the management of CV risk in patients with RA and other inflammatory joint disorders, current CV risk scores developed for the general population should be multiplied by 1.5 in subjects with RA [16]. However, most of the evidence came from RA studies, so CV risk management in other inflammatory joint disorders, including PsA, remains poorly established and whether different approaches may be needed is unknown.

The aim of this study was to compare the profile of traditional CV risk factors and MetS in patients with PsA and RA. The primary objective of our analysis was to evaluate the prevalence of MetS and its components in PsA versus RA real-world patients. Our secondary aim was to analyze the impact of MetS and its components on the occurrence of CV events in PsA versus RA real-world patients.

## 2. Materials and Methods

### 2.1. Study Design and Patients

This was a real-life observational study performed to primarily investigate the impact of MetS and its components in patients with PsA and RA referring to a Tertiary level rheumatology clinic in Italy between 2017 and 2022. The charts of all patients with PsA or RA referred to the clinic were reviewed between January 2021 and August 2021. Following data collection (baseline), patients were followed-up for an additional 12 months to gain insight into the course of MetS and its components. All patients with PsA fulfilled the 2006 Classification for Psoriatic Arthritis (CASPAR) criteria [17] and all patients with RA fulfilled the 2010 American College of Rheumatology (ACR)/EULAR criteria for RA [18]. MetS was defined according to the American Heart Association (AHA)/National Heart, Lung, and Blood Institute (NHLBI) by the presence of ≥3 of the following criteria: (1) fasting glycemia ≥ 100 mg/dL or on antihyperglycemic treatment; (2) blood pressure ≥ 130/86 mm Hg or on antihypertension treatment; (3) triglycerides ≥150 mg/dl or on treatment to lower triglycerides; (4) high-density lipoprotein cholesterol (HDL-C) < 40 mg/dl in men and <50 mg/dl in women; (5) waist circumference ≥102 cm in men and ≥88 cm in women [19]. All patients gave informed consent to participate in the study, which was conducted in accordance with the Declaration of Helsinki and local regulations. The Institutional Review Board granted an exemption of ethics committee approval due to local regulations, as the participants underwent clinical and clinimetric examinations according to routine protocols used at the recruiting hospital and no further procedures were needed.

### 2.2. Parameters Analyzed

**Figure 1:**
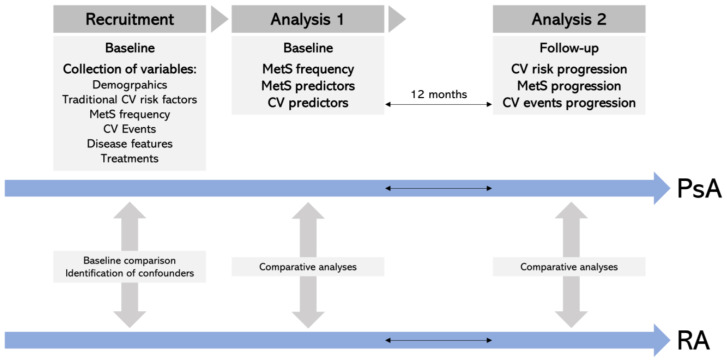
Research flowchart. Research flowchart showing the main steps (recruitment and analysis), stages (baseline and follow-up), and analysis pipeline of the present study. Gray, vertical arrows represent the main statistical analyses performed to compare RA and PsA groups according to the objectives of the study.

The following parameters were collected from patient charts: demographic and clinical characteristics (presence of autoantibodies; C-reactive protein levels; disease duration); presence of CV risk factors, including smoking, MetS, diabetes mellitus, arterial hypertension, and dyslipidemia; occurrence of CV events (ischemic heart disease, stroke, transient ischemic attack, heart failure or peripheral arterial disease). During the 12-month observation period following data extraction, the CV risk profile of the patients and the occurrence of CV events were recorded using the definitions and procedures described above. All data were anonymized before further analysis. The research process and analysis pipeline are summarized in Figure 1.

### 2.3. Statistical Analysis

Data were analyzed by descriptive statistics and variables were summarized here as absolute numbers and percentages or mean values ± standard deviation (SD), as appropriate. Differences between groups were evaluated using chi-square tests and binomial logistic regression analyses. Odds ratios (OR) and 95% confidence intervals (CI) were computed. Confounding factors were entered as covariables in the multivariate models. Statistical analysis was performed using the IBM SPSS Statistics software, version 27.0; IBM SPSS Statistics; Irving, TX, USA.

## 3. Results

### 3.1. Characteristics of PsA and RA Patients

Patients with PsA were significantly younger and had a significantly shorter disease duration than patients with RA (Table 1). As expected, the presence of autoantibodies (anti-citrullinated protein antibodies, ACPA, and rheumatoid factor, RF) was significantly higher in RA than in PsA patients (Table 1).

MetS was reported in 51.3% of PsA patients versus 27.2% of RA patients (*p* = 0.002) and dyslipidemia was reported in 71.8% of PsA patients versus 28.3% of RA patients (*p* < 0.001); these differences remained statistically significant after adjusting for the age difference (Table 1). Importantly, disease duration was not associated with MetS occurrence in RA (*p* = 0.114) nor in PsA patients (*p* = 0.234). The prevalence of the other CV risk factors considered (smoking, diabetes mellitus, hypertension) was similar in the two groups (Table 1). Moreover, the prevalence of these risk factors failed to show any association with disease duration in both conditions (Supplementary Table S1). Similar proportions of patients reported a history of CV events (28.2% of PsA patients versus 26.1% of RA patients, *p* = 0.304).

### 3.2. Association between the Presence of Autoantibodies and CV Risk Factors

A slight association between ACPA and smoking habit was found in RA (OR 2.55, 95% CI 0.65 to 6.89; *p* = 0.060), whereas there were no associations between the presence of ACPA and the other traditional risk factors. The autoantibody RF was associated in RA patients with diabetes mellitus (OR 0.43, 95% CI 0.18 to 1.02; *p* = 0.054). Autoantibodies were found to be unrelated to the occurrence of CV events in patients with RA (ACPA: *p* = 0.691, RF: *p* = 0.600). The small number of PsA patients with autoantibodies prevented the analysis of the association between autoantibody positivity and CV risk factors and events.

### 3.3. Predictors of Traditional CV Risk Factors and MetS

The occurrence of hypertension was strongly related to MetS occurrence in PsA (OR 17.88, 95% CI 5.51 to 58.00; *p* < 0.001), while it was associated with MetS (OR 11.16, 95% CI 2.99 to 41.61; *p* < 0.001), age (OR 1.09, 95% CI 1.04 to 1.14; *p* < 0.001) and dyslipidemia (OR 2.88, 95% CI 1.07 to 7.78; *p* = 0.036) in RA patients in univariate analyses. Smoking was unrelated to the other risk factors in both conditions. Dyslipidemia was slightly associated with hypertension in RA but not in PsA (Supplementary Tables S2 and S3, Supplementary Materials). Importantly, age was ruled out as a potential confounding factor in our analyses. Finally, equivalent results were observed when these associations were adjusted by treatment usage.

Univariate analyses also found a strong correlation between the occurrence of MetS and both diabetes mellitus (OR 20.71, 95% CI 4.34 to 98.78; *p* < 0.001) and hypertension (OR 17.88, 95% CI 5.51 to 57.98; *p* < 0.001) in PsA. The main predictors of MetS in RA, according to univariate analysis, were dyslipidemia (OR 18.73, 95% CI 5.77 to 60.81; *p* < 0.001) and hypertension (OR 11.16, 95% CI 2.99 to 41.61; *p* < 0.001). Multivariate analysis confirmed the differences between PsA and RA in terms of MetS predictors (Table 2): hypertension and dyslipidemia were MetS predictors in PsA, while diabetes mellitus, hypertension, and dyslipidemia were MetS predictors in RA. Notably, hypertension and dyslipidemia were the strongest MetS predictors in PsA and RA, respectively, (Table 2). Of note, age was found not to be a significant predictor in any condition, thus ruling out a major confounding effect. Similarly, adjusting for disease duration did not attenuate these associations (Table 2). Finally, adjusting for NSAIDs or anti-TNF agents did not change these results (Tables S4 and S5, Supplementary Materials). Equivalent results were observed for the rest of medications.

All these results suggest not only that not all MetS components were consistently associated with MetS presence, but also that significant differences in predictors as well as their strength of associations were observed across conditions.

### 3.4. Predictors of CV Events

Univariate analysis showed that MetS and some of its components (diabetes mellitus and hypertension) accounted for the history of CV events in PsA [OR (95% CI); MetS: 34.83 (4.30 to 281.99); diabetes mellitus: 7.99 (2.61 to 24.41); and hypertension: 31.00 (3.87 to 284.43); *p* < 0.001 for all associations]. In RA, MetS was also a predictor of CV events (OR 5.20, (1.84 to 14.70); *p* = 0.001), but the predictive components of MetS with statistical significance were hypertension (OR 4.27, 95% CI 1.43 to 12.77; *p* = 0.007) and dyslipidemia (OR 5.00, 95% CI 1.83 to 13.66; *p* < 0.001), while diabetes mellitus was not significantly associated with CV events. The associations were markedly stronger in PsA than in RA. Multivariate analysis confirmed these associations (Table 3): hypertension and MetS were the only predictors of CV events in PsA, while dyslipidemia was the only factor to be significantly associated with CV events in RA. Again, disease duration failed to show any association in both conditions (Table 3). Finally, equivalent findings were observed after adjusting for treatments (Table S6, Supplementary Material).

The fact that the association of traditional risk factors and MetS with CV events was stronger in PsA than in RA was corroborated by the greater coefficient of determination in PsA than in RA, indicating that in PsA approximately 70% of the variance in CV events could be explained by the variables entered in the model, compared with only 30% in RA. Adjusting for treatments did not attenuate these coefficients, thus ruling out a major effect of medications and pointing to a role of disease characteristics.

### 3.5. Outcomes after 12 Months

To evaluate the potential progression of MetS and its components in PsA and RA in the real-world setting, patients were followed up for 12 months, following recruitment in the study. Changes in the occurrence of CV risk factors and MetS over the 12-month follow-up of both PsA and RA patients were negligeable (Table 4).

## 4. Discussion

The present study confirms that several differences exist between PsA and RA in the profile of traditional CV risk factors leading to MetS occurrence and the association of these factors with the history of CV events. According to our findings, patients with PsA had a significantly higher prevalence of MetS and dyslipidemia compared to patients with RA, while the frequency of other traditional CV risk factors was comparable. Notably, the rates of CV events reported before data collection were relatively high in both groups (>25%) as expected, but similar. PsA and RA were also found to differ in terms of predictors of MetS: hypertension was the strongest predictor in PsA, while in RA the strongest predictor was dyslipidemia. Furthermore, MetS and hypertension were both shown to be significantly associated with a history of CV events in patients with PsA; in RA, only dyslipidemia was confirmed as a predictor of CV events in a multivariate model, despite exhibiting a lower frequency compared to PsA patients. Importantly, these findings remained after adjusting for age and disease duration, thereby excluding a major confounding role and pointing towards a role for disease characteristics in determining these associations. Moreover, adjusting for treatment usages did not change these associations, hence reinforcing this notion. Furthermore, equivalent differences in the profiles of associations were observed in a well characterized prospective cohort from Spain comparing RA, PsA and ankylosing spondylitis patients [20]. Taken together, these findings suggest that, although proof-of-concept, quantitative and qualitative differences regarding MetS presence could occur between RA and PsA, with potential impact on CV records across different arthropathies in real-world populations.

Several authors have investigated the role of traditional CV risk factors in chronic inflammatory conditions, with most of the efforts being devoted to RA. A study comparing the incidence of CV events in patients with RA versus the general population confirmed the higher incidence in RA [21]. After adjusting for traditional risk factors, the incidence decreased only slightly suggesting that other mechanisms are responsible for the increased rates of CV events in RA patients. Systemic inflammation, known from other studies to be involved in the development of atherosclerosis, was suggested as a possible contributor [21]. In a subsequent study, three groups of variables were tested for their effects on carotid atherosclerosis in RA, namely demographic, traditional CV risk factors, and RA manifestations [22]. The study found that demographic characteristics (age and gender) best explained the variability of atherosclerosis. The study also showed that, after adjusting for the effects of age and gender, both traditional CV risk factors and RA manifestations had a greater impact on atherosclerosis [22]. This, along with the evidence from other studies, suggested that traditional CV factors and inflammation may interact and promote atherogenesis [22,23]. An elegant study put this association into numbers, reporting that traditional CV risk factors accounted for 49% of the total risk and non-traditional risk factors (RA manifestations, inflammation, etc.) accounted for a smaller proportion (30.3%) [24]. Our results align with this evidence and may expand our knowledge about the complex interplay of CV risk factors in chronic inflammatory diseases. Traditional CV risk factors, according to the available evidence, account only for a limited fraction of the variance, which suggests the participation of other risk factors. Our findings show that MetS was independently associated with a history of CV events in multivariate models, at least in PsA, suggesting that MetS occurrence may have an additional impact, beyond that of its individual components, probably due to the existence of synergistic effects. Our findings revealed that MetS is present in a significant proportion of established PsA patients, which emphasizes the need of systematically screening and managing MetS in arthropathies. In fact, a recent meta-analysis reported a prevalence of MetS in PsA populations of 29.1%, ranging from 23.5% to 62.9% in the literature, which is in line with our cohort. Although a slightly lower prevalence was observed in a recent cohort from Spain (30.6%) [20,25], it must be noted that our cohort exhibited a higher frequency of dyslipidemia, which may explain the higher prevalence herein reported. As a consequence, MetS should be considered as a separate entity in clinical practice and routine screening of CV risk factors, but this situation may be different in different joint diseases. In this regard, it is tempting to speculate if different presentations of MetS can lead to different endotypes or pathotypes that require distinct management strategies, including screening and treatment algorithms. This may facilitate the take-decision process in the clinical setting and ensure a tailored clinical process. In fact, personalized medicine approaches have been already suggested for MetS [26,27,28,29], which may strengthen this notion.

In our analysis, the strongest predictor of CV events in patients with RA was dyslipidemia, which is known to be largely influenced by inflammation in RA [24,30]. Similar results were obtained a large, prospective Spanish cohort [25]. Importantly, this association was stronger in RA compared to PsA, despite the high prevalence in the latter, hence pointing to a relevance that may not be captured by its conventional definition/approach. It can therefore be speculated that the strong association observed may be caused, at least in part, by the lipid-inflammation crosstalk. The interaction of systemic inflammation with traditional CV risk factors is not fully understood. In RA, the evidence shows that systemic inflammation can modify traditional risk factors, especially lipid levels [3]. For example, in active RA, inflammation has been shown to modulate lipid and lipoprotein metabolism and to be associated with lower levels of lipids [3,24,30,31,32,33]. However, the decreased levels of lipids are unexpectedly associated with an increased CV risk, which is known as the “lipid paradox” [34]. A possible explanation of this paradox is that inflammation modifies low-density lipoprotein cholesterol (LDL-C) through oxidation; oxidized LDL-C is atherogenic resulting in increased CVD risk despite low serum levels [3]. However, other functions, such as enzymatic activities, pro-oxidant mechanisms, humoral response activation or pathways involving acute-phase mediators may be also implicated. In fact, the preventive effect of HDL-C on LDL-C oxidation has been shown to be impaired in RA patients compared with controls [32]. Taken together, these lines of evidence have led to a paradigm shift from the “lipid paradox” to the concept of HDL dysfunction [35]. However, HDL dysfunction phenomena in chronic inflammatory diseases still need to be thoroughly characterized [34,35]. The findings presented herein underline the central role of lipids and dyslipidemia in RA as a key factor to understand CV history, even in the presence of MetS or other traditional risk factors.

With regards to CV risk factors associated with PsA, the available evidence shows that metabolic comorbidities, obesity in particular, are involved [14,15,35]. The distinction in terms of CV risk profile from RA, despite the fact that both diseases are characterized by chronic inflammation, has been previously addressed [8,9,35,36]. It has been pointed out that, contrary to RA where systemic inflammation appears to contribute directly to the increased CV risk, in PsA traditional risk factors, including adiposity and MetS, are major contributors to the CV risk [8,35,37]. These differences were also reported in a recent study [38], although the impact of MetS was not explored. Our findings showing a significantly higher prevalence of MetS and dyslipidemia in PsA versus RA patients (despite the fact that the PsA population was significantly younger than the RA population) and the stronger association of MetS with a history of CV events in patients with PsA strongly reinforce these observations, also highlighting the differential contribution of MetS. Again, these associations were independent of disease duration, which is thought to have a different impact in RA and PsA, as evidence of a causal role is less robust in the latter [20,38,39]. Further research is needed to evaluate the impact of disease progression on MetS in the long term in inflammatory arthropathies.

During the 12-month follow-up, the prevalence of MetS and its components in both patient groups was markedly stable. This suggests that in established RA and PsA, provided that no disease flares and major treatment changes occur, the screening of CV risk factors and MetS can be performed on a yearly basis, as recommended by current EULAR guidelines [16]. Whether more frequent screening may be required in early disease or in specific patient populations (elderly, patients with comorbidities) needs to be investigated.

The fact that distinct factors are involved in the increased CV risk in different chronic inflammatory diseases raises the question as to what extent the prevention and management of CV risk should be disease specific. Compelling evidence confirms that early diagnosis and treatment may reduce the frequency of CV events and improve survival in patients with arthropathies [40]. Current practice is based on general recommendations for RA and “other forms of inflammatory joint disorders”, with most of the evidence on which the recommendations are based coming from studies in patients with RA [16]. In patients with RA, it may be crucial to reduce disease activity and systemic inflammation, beside reducing traditional CV risk factors with a special focus on lipids [1,38,41]. A better understanding of this crosstalk will warrant optimal CV management. Patients with PsA, on the other hand, may benefit more from broader CV-prevention strategies that target MetS and hypertension [36]. Further research is needed to explore the feasibility and implementation in clinical practice, as well as the cost-effectiveness of disease-specific versus general recommendations for CV prevention.

Our study has several limitations including the retrospective design and the small sample size of both populations. The two populations compared were not matched in terms of mean age and disease duration; this was due to the fact that RA and PsA have a different pathogenesis and age of onset, and probably attending/organizational variables. No strict clinical features were used as inclusion/exclusion criteria in order to capture real-world populations and avoid highly selected patient samples. Therefore, this potential limitation is hard to avoid in unselected populations that are representative of patients encountered in real-life, although extensive analytical steps were performed to correct for these differences. Of note, none of these variables were observed to show a strong confounding effect. Our findings can be considered as proof-of-concept and pave the ground for future larger and prospective trials assessing the impact of MetS on inflammatory arthropathies. Furthermore, time-adjusted risk factor exposure analyses are needed to evaluate the real impact of MetS and its components.

## 5. Conclusions

The study shows that MetS and its components differently impact the risk of CVD in PsA and RA. The clinical presentation of MetS may differ between PsA and RA. These findings are relevant for clinical practice as a disease-specific management of CV risk may be required in distinct chronic inflammatory diseases of the joints. However, clinical validation in larger studies is needed. Further efforts are required to develop disease-specific strategies for the management of CV risk in PsA and RA.

## Figures and Tables

**Table 1:** Demographic and clinical characteristics of patients.

	PsA (n = 78)	RA (n = 92)	*p*-Value	*p*-Value (Age-Adjusted)
**Demographic features**				
Age, years, mean (±SD)	45.23 (16.99)	60.81 (13.19)	<0.001	
Sex, n (%)				
Female	54 (69.2)	70 (76.1)	0.316	
Male	24 (30.8)	22 (23.9)		
**Clinical features**				
RF, n (%)	6 (7.7)	54 (58.7)	<0.001	
ACPA, n (%)	4 (5.1)	53 (57.6)	<0.001	
CRP, mg/L, mean (±SD)	5.14 (7.91)	6.63 (9.21)	0.047	
Disease duration, months, mean (±SD)	18.86 (48.43)	176.85 (169.77)	<0.001	
**Treatments**				
Steroids, n (%)	12 (15.4)	37 (40.2)		
NSAIDs, n (%)	39 (50.0)	0		
Methotrexate, n (%)	13 (16.7)	44 (47.8)		
Leflunomide, n (%)	3 (3.8)	7 (7.6)		
Sulfasalazine, n (%)	5 (6.4)	4 (4.3)		
Anti-TNF, n (%)	49 (62.8)	23 (29.5)		
Anti-IL6-R, n (%)	0	8 (8.7)		
Abatacept, n (%)	0	32 (34.8)		
JAK inhibitors, n (%)	0	6 (6.5)		
Anti-CD20, n (%)	0	2 (2.2)		
Apremilast, n (%)	5 (6.4)	0		
Anti-IL17, n (%)	10 (12.8)	0		
Anti-IL12, n (%)	3 (3.8)	0		
Anti-IL23, n (%)	1 (1.3)	0		
**Traditional CV risk factors**				
Smoking, n (%)	22 (28.2)	26 (28.3)	0.836	0.997
MetS, n (%)	40 (51.3)	25 (27.2)	0.003	0.002
Diabetes mellitus, n (%)	26 (38.3)	33 (35.9)	0.823	0.516
Hypertension, n (%)	44 (56.4)	51 (55.4)	0.749	0.013
Dyslipidemia, n (%)	56 (71.8)	26 (28.3)	<0.001	<0.001
**History of CV events**				
CV events ^a^, n (%)	22 (28.2)	24 (26.1)	0.602	0.304

^a^ Composite of ischemic heart disease, stroke, transient ischemic attack, heart failure, or peripheral arteriopathy reported in the charts of PsA and RA patients. ACPA, anti-citrullinated protein anti bodies; CRP, C-reactive protein; CV, cardiovascular; MetS, metabolic syndrome; PsA, psoriatic arthritis; RA, rheumatoid arthritis; RF, rheumatoid factor.

**Table 2:** Predictors of MetS occurrence in patients with PsA and RA. Multivariate analysis using an age-adjusted logistic regression model; the coefficient of determination R^2^ of each model is indicated.

	OR (95% CI)	*p*-Value	OR (95% CI) [Adjusted]	*p*-Value [Adjusted]
**PsA (R^2^ = 0.543)**				
Age	0.979 (0.929–1.033)	0.445	0.981 (0.933–1.033)	0.468
Sex	0.693 (0.153–3.142)	0.634	0.565 (0.121–2.634)	0.467
Smoking	2.041 (0.442–9.429)	0.361	2.626 (0.459–15.029)	0.278
Diabetes mellitus	6.465 (0.952–43.899)	0.056	6.638 (0.899–49.545)	0.065
Hypertension	11.818 (2.046–58.053)	0.002	10.457 (1.853–59.007)	0.002
Dyslipidemia	5.190 (1.118–24.092)	0.035	5.190 (1.383–49.273)	0.021
**RA (R^2^ = 0.669)**				
Age	0.976 (0.914–1.043)	0.473	0.978 (0.915–1.046)	0.519
Sex	1.568 (0.272–9.041)	0.615	1.466 (0.250–8.583)	0.672
Smoking	0.404 (0.068–2.387)	0.317	0.395 (0.067–24487)	0.307
Diabetes mellitus	15.586 (2.418–100.453)	0.004	13.709 (2.057–91.389)	0.007
Hypertension	20.447 (2.589–161.057)	0.004	18.676 (2.359–147.859)	0.006
Dyslipidemia	43.296 (6.823–274.756)	<0.001	39.039 (6.068–251.159)	<0.001

CI, confidence interval; OR, odds ratio; RA, rheumatoid arthritis; PsA, psoriatic arthritis. OR (95% CI) [adjusted] and *p*-value [adjusted]: OR and *p*-values obtained in a multivariate analysis adjusted by disease duration.

**Table 3:** Predictors of cardiovascular events at baseline. Multivariate analysis using an age-adjusted logistic regression model; the coefficient of determination R^2^ of each model is indicated. Baseline is defined as the time of data collection.

	*p*-Value	OR (95% CI)
**PsA (R^2^ = 0.693)**		
Smoking	0.154	3.884 (0.602–25.058)
MetS	0.031	22.611 (1.323–58.655)
Diabetes mellitus	0.250	0.276 (0.031–2.468)
Hypertension	0.010	50.302 (2.583–97.661)
Sex	0.445	0.504 (0.087–2.921)
Dyslipidemia	0.241	3.225 (0.455–22.879)
CRP	0.560	0.950 (0.799–1.129)
Age	0.740	0.986 (0.910–1.069)
Disease duration	0.134	0.965 (0.921–1.011)
**RA (R^2^ = 0.296)**		
Smoking	0.298	1.896 (0.568–6.239)
MetS	0.702	1.358 (0.282–6.531)
Diabetes mellitus	0.413	1.896 (0.568–6.329)
Hipertensión	0.129	3.004 (0.726–12.434)
Sex	0.586	0.586 (0.174–2.688)
Dyslipidemia	0.011	4.502 (1.118–16.125)
CRP	0.518	1.020 (0.961–1.081)
Age	0.669	1.012 (0.960–1.066)
Disease duration	0.329	0.997 (0.992–1.003)

CI, confidence interval; CRP, C-reactive protein; OR, odds ratio; RA, rheumatoid arthritis; PsA, psoriatic arthritis.

**Table 4:** Changes in the occurrence of CV risk factors and CV events in patients with PsA and RA observed for 12 months. Baseline is defined as the time of data collection.

***Presence of CV Risk Factors, n(%)***			
	**Baseline**	**12-Month Follow-Up**	***p*-Value**
**Patients with PsA (N = 78)**			
MetS	40 (51.3)	41 (52.6)	0.207
Diabetes mellitus	26 (33.3)	26 (33.3)	
Hypertension	44 (56.4)	43 (55.1)	0.159
**Patients with RA (N = 92)**			
MetS	25 (27.2)	25 (27.2)	
Diabetes mellitus	33 (35.9)	33 (35.9)	
Hypertension	51 (55.4)	51 (55.4)	
***Occurrence of CV Events, n(%)***			
	**Baseline**	**12-Month Follow-Up**	***p*-Value**
**Patients with PsA (N = 78)**			
Ischemic heart disease	13 (16.7)	13 (16.7)	
Arrhythmia/Atrial fibrillation	12 (15.4)	8 (10.3)	0.023
Stroke	4 (5.1)	4 (5.1)	
Transient ischemic attack/Peripheral arterial disease	18 (23.1)	18 (23.1)	
**Patients with RA (N = 92)**			
Ischemic heart disease	8 (8.7)	8 (8.7)	
Arrhythmia/Atrial fibrillation	4 (4.3)	4 (4.3)	
Stroke	4 (4.3)	4 (4.3)	
Transient ischemic attack/Peripheral arterial disease	8 (8.7)	8 (8.7)	

CV, cardiovascular; MetS, metabolic syndrome; PsA, psoriatic arthritis; RA, rheumatoid arthritis.

## Data Availability

The detailed data will be available on request from the corresponding author.

## Supplementary Materials

The following supporting information can be downloaded at https://www.mdpi.com/article/10.3390/jcm12155031/s1: Table S1: Associations between CV risk factors and disease duration; Table S2: Predictors of smoking; Table S3: Predictors of dyslipidemia; Table S4: Predictors of MetS oc-currence adjusted for NSAIDs; Table S5: Predictors of MetS occurrence adjusted for anti-TNF; Table S6: Predictors of CV events at baseline.
