# Supplementary material for: Metabolic Syndrome and Its Components Have a Different Presentation and Impact as Cardiovascular Risk Factors in Psoriatic and Rheumatoid Arthritis
Source: J Clin Med. 2023 Jul 31;12(15):5031. doi: 10.3390/jcm12155031 (PMC10420311; doi:10.3390/jcm12155031)
Supplement: Supplementary file 1 [file jcm-12-05031-s001.zip › jcm-2515844-supplementary.pdf]

Supplementary Material

Table S1. Associations between CV risk factors and disease duration. Variables were summarized as mean ± SD. Differences were assessed by Mann-Withney U tests.

	No	Yes	p-Value
**PsA**			
Diabetes mellitus	27.34 ± 58.16	18.57 ± 17.55	0.135
Hypertension	35.36 ± 68.62	25.52 ± 21.19	0.119
Smoking	18.41 ± 52.30	21.90 ± 40.56	0.250
Dyslipidemia	18.86 ± 48.43	17.25 ± 14.41	0.626
**RA**			
Diabetes mellitus	189.89 ± 113.64	154.09 ± 115.90	0.256
Hypertension	157.22 ± 96.41	142.25 ± 180.02	0.854
Smoking	141.00 ± 90.00	149.00 ± 100.16	0.535
Dyslipidemia	176.85 ± 169.77	125.56 ± 115.28	0.635

Table S2. Predictors of smoking. Univariate analysis; p-values were estimated using the chi-squared test.

	p-Value	OR (95% CI)
**PsA**		
Age	0.694	0.993 (0.962 – 1.026)
Sex	0.646	0.772 (0.256 – 2.326)
Rheumatoid factor	0.271	2.579 (0.478 – 13.916)
ACPA	0.386	2.450 (0.322 – 18.613)
MetS	0.910	0.943 (0.342 – 2.602)
Diabetes mellitus	0.774	0.856 (0.295 – 2.482)
Hypertension	0.487	0.700 (0.256 – 1.912)
Dyslipidemia	0.243	0.525 (0.178 – 1.549)
**RA**		
Age	0.973	1.011 (0.966 – 1.036)
Sex	0.671	1.253 (0.443 – 3.545)
Rheumatoid factor	0.083	2.404 (0.891 – 6.485)
ACPA	0.064	2.555 (0.947 – 6.889)
MetS	0.782	1.108 (0.531 – 2.315)
Diabetes mellitus	0.745	1.168 (0.457 – 2.986)
Hypertension	0.263	0.593 (0.238 – 1.480)
Dyslipidemia	0.397	1.526 (0.574 – 4.059)

ACPA — anti-citrullinated protein antibodies; CI — confidence interval; MetS — metabolic syndrome; OR — odds ratio; PsA — psoriatic arthritis; RA — rheumatoid arthritis.

Table S3. Predictors of dyslipidemia. Univariate analysis; p-values were estimated using the chi-squared test.

	**p-Value**	**OR (95% CI)**
**PsA**		
Smoking	0.239	0.525 (0.178 – 1.549)
MetS	0.081	2.571 (0.876 – 7.549)
Diabetes mellitus	0.609	1.333 (0.443 – 4.017)
Hypertension	0.778	0.861 (0.304 – 2.439)
Sex	0.422	0.650 (0.226 – 1.866)
Rheumatoid factor	0.684	0.691 (0.117 – 4.105)
ACPA	0.215	1.078 (1.001 – 1.161)
CRP	0.236	1.097 (0.941 – 1.280)
Age	0.422	1.015 (0.978 – 1.054)
**RA**		
Smoking	0.396	1.526 (0.574 – 4.059)
MetS	< 0.001	18.735 (5.772 – 60.812)
Age	0.408	1.015 (0.979 – 1.053)
Sex	0.510	0.686 (0.224 – 2.104)
Rheumatoid factor	0.201	1.875 (0.715 – 4.914)
ACPA	0.345	1.574 (0.614 – 4.038)
Diabetes mellitus	0.523	0.729 (0.276 – 1.923)
Hypertension	0.036	2.884 (1.069 – 7.777)
CRP	0.843	1.005 (0.958 – 1.055)

ACPA — anti-citrullinated protein antibodies; CI — confidence interval; CRP — C-reactive protein; MetS — metabolic syndrome; OR — odds ratio; PsA — psoriatic arthritis; RA — rheumatoid arthritis.

Table S4. Predictors of MetS occurrence adjusted for NSAIDs. Multivariate analysis adjusted by disease duration and NSAIDs usage; the coefficient of determination (R2) of each model (PsA and RA) is indicated).

	**OR (95% CI)**	**p-Value**
**PsA (R^2 = 0.601)**		
Age	0.994 (0.946 – 1.045)	0.761
Sex	0.843 (0.190 – 3.768)	0.619
Smoking	1.947 (0.399 – 9.494)	0.410
Diabetes mellitus	5.325 (0.796 – 38.032)	0.085
Hypertension	14.688 (2.683 – 80.418)	0.002
Dyslipidemia	4.940 (1.008 – 22.234)	0.041
**RA (R^2 = 0.669)**		
Age	0.976 (0.914 – 1.043)	0.473
Sex	1.568 (0.272 – 9.041)	0.615
Smoking	0.404 (0.068 – 2.387)	0.317
Diabetes mellitus	15.586 (2.418 – 100.453)	0.004
Hypertension	20.447 (2.589 – 161.057)	0.004
Dyslipidemia	43.296 (6.823 – 274.756)	<0.001

CI — confidence interval; MetS — metabolic syndrome; OR — odds ratio; PsA — psoriatic arthritis; RA — rheumatoid arthritis.

Table S5. Predictors of MetS occurrence adjusted for anti-TNF. Multivariate analysis adjusted by disease duration and anti-TNF usage; the coefficient of determination (R2) of each model (PsA and RA) is indicated).

	OR (95% CI)	p-Value
**PsA (R^2 = 0.560)**		
Age	0.977 (0.925 – 1.031)	0.396
Sex	0.566 (0.116 – 2.669)	0.563
Smoking	2.097 (0.453 – 9.705)	0.343
Diabetes mellitus	7.049 (0.997 – 49.831)	0.051
Hypertension	13.151 (2.259 – 68.377)	0.002
Dyslipidemia	5.945 (1.208 – 29.256)	0.028
**RA (R^2 = 0.669)**		
Age	0.913 (0.900 – 1.215)	0.313
Sex	1.516 (0.178 – 9.002)	0.570
Smoking	0.354 (0.054 – 2.145)	0.289
Diabetes mellitus	16.781 (2.012 – 95.114)	0.009
Hypertension	21.145 (2.123 – 91.112)	0.003
Dyslipidemia	41.122 (5.932 – 195.574)	<0.001

CI — confidence interval; MetS — metabolic syndrome; OR — odds ratio; PsA — psoriatic arthritis; RA — rheumatoid arthritis.

Table S6. Predictors of CV events at baseline. Multivariate analyses adjusted by NSAIDs (model 1), anti-TNF (model 2) or steroids (model 3) usage were performed, and corresponding p-values are shown in the table for each model and risk factor.

	p-Value (model 1)	p-Value (model 2)	p-Value (model 3)
**PsA**			
Smoking	0.092	0.177	0.156
MetS	0.014	0.031	0.030
Diabetes mellitus	0.235	0.256	0.229
Hypertension	0.016	0.010	0.009
Sex	0.090	0.460	0.498
Dyslipidemia	0.082	0.247	0.225
CRP	0.125	0.092	0.209
Age	0.267	0.740	0.785
Disease duration	0.086	0.151	0.116
**RA**			
Smoking	0.298	0.201	0.301
MetS	0.702	0.312	0.258
Diabetes mellitus	0.413	0.241	0.358
Hypertension	0.129	0.090	0.163
Sex	0.586	0.351	0.361
Dyslipidemia	0.011	0.021	0.019
CRP	0.518	0.314	0.351
Age	0.669	0.788	0.654
Disease duration	0.329	0.452	0.311

CI — confidence interval; CRP — C-reactive protein; MetS — metabolic syndrome; OR — odds ratio; PsA — psoriatic arthritis; RA — rheumatoid arthritis.
